# Adrenalin by Unusual Routes

**Published:** 1917-01

**Authors:** 


**Adrenalin by Unusual Routes.** The Ther. Gaz., edit., Oct., mentions resuscitation of a child by injection of adrenalin into the umbilic stump, and cites experiments by Auer & Gates (Jour. Exp. Med., June), showing that intratracheal injections promptly influence blood pressure in rabbits. Leland Boogher of St. Louis reported in the N. Y. Med. Jour., last summer the use of adrenalin per os, which prompted us to confess that we were another one to use this drug in the same way, in spite of the almost universal denunciation of anything short of hypodermic administration.

